# 

**Published:** 1890-08-30

**Authors:** 


### NOTICE TO CORRESPONDENTS.

All MS., Letters, Books for Review, and other matters intended for the Editor should be addressed The Editor, The Lodge, Porchester Square, London, W.

The Editor cannot undertake to return rejected M.S., even when accompanied by stamped directed envelope.

**Notice to Advertisers and Subscribers.**

All Advertisements, Orders for Copies of The Hospital, and Business Communications should be addressed to The Publisher, *not to the Editor*, The Hospital, 140, Strand, W.C.

**Bound Volumes of "The Hospital."**

Vol. VI., containing full page portraits of T.R.H. the Prince and Princess of Wales, and Miss Florence Nightingale, is obtainable at 4s., post free.

Orders will be received for Vol. VII., 4s. post free. Cases for binding may be had of the Publisher, at 1s. 6d. each.

**To Residents, Secretaries, and Matrons.**

The Editor will be glad to have the Regulations and each Report as issued by Asylums, Hospitals, Convalescent and Nursing Institutions, as well as those of other Charities, and an early copy in duplicate of all Appeals and Festival Notices, etc., addressed to *The Editor, The Lodge, Porchester Square, London, W.* Any superintendent, secretary, matron, or other chief official who regularly sends such information may be registered, on application to the Publisher, to receive free of cost a cover for binding The Hospital in half-yearly volumes.

### BURDETT'S HOSPITAL ANNUAL FOR 1890

Is now ready, and may be obtained of the Publisher, The Hospital Offices, 140, Strand, W.C., price 2s. 6d. limp boards, or 3s. 6d. cloth. All orders must be accompanied by a remittance.

### Scraps and Gleanings.

Two children were killed by cricket balls lately.

The Autumn Congress of the Sanitary Institute meets this week at Brighton.

Hertford kept Hospital Sunday with a very grand parade. Over £100 was collected.

The death is announced at University College Hospital of Mr. J. A. C. Morrison, of Singapore.

The Corporation of Salford has sent to M. Pasteur two children who were last week bitten by a mad dog.

The Shah of Persia has telegraphed for his physician, Dr. Tholosan, because he feels so lonely without him.

An examination for seventeen appointments as surgeon in the Indian medical service will be held in February next.

The *Daily News* of August 18th contained a long account of the Retreat for Inebriate Women at Fallowfield, Manchester.

Mrs. St. A. Horton has resigned her post of residential manager and secretary to the St. Raphael's Hospital at Worthing.

Mr. Wynne E. Baxter lately held an inquest on the body of a man who died from choleraic diarrhœa brought on by eating pickles.

Eastbourne Homœopathic Convalescent Home held its annual meeting lately. The attendance was small, but the report presented was good.

Fourteen thousand pounds have been subscribed towards the new Royal Hospital which was opened at Harrogate by the Duke of Clarence last year.

Brighton is going to keep Hospital Saturday on the 30th on a very grand scale. The Mayor is busy organizing a series of dramatic entertainments.

A gymnast died at Poplar with tetanic symptoms, supposed to have been brought on by the morbid condition of his spine consequent on his occupation.

The Clothworkers' Company have sent £25 to the Hon. Alfred Lyttelton (10, Buckingham Street, Strand), for the Children's Country Holidays Fund.

Dr. Benjamin Ward Richardson, F.R.S., has been elected President of the Association of Public Sanitary Inspectors in succession to the late Sir Edwin Chadwick, K.C.B.

The Cannibals of Dubanghi are a delightfully simple race. When Father Augonard told them it was horrible to eat human flesh, they replied "No, it is delicious with salt and spices."

If a residential medical officer should be changed every five years because he becomes a nuisance, what happens if he remains thirty-five years? This query is sent to us by a contributor. Perhaps Sir Andrew Clarke can answer.

Lady Roberts's Nursing Home in the hills in India is doing good work, as the report just issued demonstrates. After paying a large sum towards building the home at Quetta, a substantial balance is left. Fresh nurses, volunteers from England, are expected out shortly.

The village of Langtoft, in Yorkshire, has lately been attacked by scarlet fever, and the medical officer of health reports that nearly all the female population have been afflicted with the disease. Only one death has occurred. Within the last twelve months there have been outbreaks of measles, mumps, influenza, and scarlatina in the village. The unhealthy state of the village is attributed to the village pond.

The Lincolnshire police arrested lately an Irishman, named Mace, for stealing spirits at an inn at Whaplode. The prisoner, however, died a few minutes after being received into the police cell at Spalding. He had lain in a cornfield for two days and nights drinking brandy and whisky, which he had stolen.

Dr. Robert Stevenson, an American physician, who visited Vienna after leaving the Medical Congress at Berlin, died suddenly in the former city on Sunday. A post mortem examination proved death to have resulted from hemorrhage of the brain. Documents in the doctor's possession show him to have come from Adrian, in the State of Michigan.

A serious outbreak of typhoid fever is reported from Murstow, near Sittingbourne, the number of cases increasing, in spite of sanitary precautions. The local infection hospital is filled with patients. The fever is supposed to have originated through the drinking by some brickfield labourers of water from a shallow well which was contaminated with sewage.

Thousands of poor children will, in accordance with the direct orders of the Emperor William, enjoy for the first time in their lives the benefit of a change to the seaside. The children of all the workmen employed in the Government factories have been examined by medical men, and such as require sea breezes will be sent for a holiday at the expense of the Government.

The idea, generally current, that death, following on disease, most frequently occurs during the early hours of morning, has been proved by a recent investigation to be erroneous. Dr. Burns, of the Charity Hospital, New York, has made observations in some 15,000 cases, extending over twelve years, with the result of finding that no particular hour or period of the day is specially fatal.

According to the *Figaro*, the prison at Sparta is in a state of revolution. A prisoner had obtained leave to keep his dog. The animal, "to gain his private ends, went mad and bit" six convicts and eleven soldiers. The Greek Government has determined to send the seventeen persons bitten to Paris to undergo the Pasteur treatment. The six convicts are to be sent at the expense of the State. One of them has raised the question whether the time spent on the journey will count as part of his penalty.

The following report is by Mr. McNeil, visiting officer to the Board of Supervision, anent the New Hospital, Paisley:—On July 21st I inspected the Abbey Parish Hospital for the first time since its completion. The wards are airy, and the ventilation, so far, has been successful. The Nursing Staff appears to be satisfactory; and generally the arrangements seem such as I would approve. Structurally, I doubt whether Great Britian shows another hospital of the same class surpassing this building in its plan.

